# The Effect of Hydrogen Sulfide on Different Parameters of Human Plasma in the Presence or Absence of Exogenous Reactive Oxygen Species

**DOI:** 10.3390/antiox8120610

**Published:** 2019-12-03

**Authors:** Beata Olas, Paulina Brodek, Bogdan Kontek

**Affiliations:** Department of General Biochemistry, Faculty of Biology and Environmental Protection, University of Lodz, Pomorska 141/3, 90-236 Lodz, Poland; paulina.brodek@gmail.com (P.B.); bogdan.kontek@biol.uni.lodz.pl (B.K.)

**Keywords:** hydrogen sulfide, plasma, oxidative stress, pro-oxidant, hemostasis

## Abstract

The main aim of the study is to examine the effect of sodium hydrosulfide (NaHS), an H_2_S donor, on the oxidative stress in human plasma in vitro. It also examined the effects of very high concentrations of exogenous hydrogen sulfide on the hemostatic parameters (coagulation and fibrinolytic activity) of human plasma. Plasma was incubated for 5–30 min with different concentrations of NaHS from 0.01 to 10 mM. Following this, lipid peroxidation was measured as a thiobarbituric acid reactive substance (TBARS) concentration and the oxidation of amino acid residues in proteins was measured by determining the amounts of thiol groups and carbonyl groups. Hydrogen peroxide (H_2_O_2_) and the hydroxyl radical generating oxidation system (Fe/H_2_O_2_) were used as oxidative stress inducers. Hemostatic factors, such as the maximum velocity of clot formation, fibrin lysis half-time, the activated partial thromboplastin time (APTT), thrombin time (TT), and international normalized ratio (INR), were estimated. Changes in lipid peroxidation, carbonyl group formation, and thiol group oxidation were detected at high concentrations of H_2_S (0.1–10 mM), and these results indicate that NaHS (as the precursor of H_2_S) may have pro-oxidative effects in human plasma in vitro. Moreover, considering the data presented in this study, we suggest that the oxidative stress stimulated by NaHS (at high concentrations: 1–10 mM) is not involved in changes of the hemostatic activity of plasma.

## 1. Introduction

Hydrogen sulfide (H_2_S) is a well-known toxic gas synthesized from the amino acids l- and d-cysteine (Cys) and l-homocysteine (l-Hcy). Its biosynthesis involves four enzymes: cystathionine-β-synthase (CBS), mercaptopyruvate sulfurtransferase (3-MST), cystathionine-γ-lyase (CSE), and cysteine aminotransferase [1]. H_2_S is known to play an important role in various biological systems, including the cardiovascular system [2,3,4,5,6,7,8], and our own previous research has shown that the administration of 0.01 and 0.1 mM H_2_S may reduce the plasma lipid peroxidation induced by various forms of homocysteine [9], which indicates the existence of a relationship between the presence of homocysteine and cardiovascular diseases. 

Zaicho et al. [10] classified modulators of H_2_S metabolism into three groups: (1) agents that reduce the amount of hydrogen sulfide in tissues (specific and nonspecific inhibitors of H_2_S synthesis), (2) agents with an uncertain impact (some medicines), and (3) agents that increase the amount of hydrogen sulfide (inorganic and organic H_2_S donors). Sodium hydrosulfide (NaHS) and sodium sulfide (Na_2_S) were the first H_2_S donors to be studied in the cardiovascular system [11]. Na_2_S is currently under evaluation in phase I and II trials as a therapeutic agent. 

As the influence of H_2_S on oxidative stress remains unknown, and is sometimes controversial, the aim of our study was to determine the effects of sodium hydrosulfide on oxidative stress in human plasma when administered at concentrations of 0.01 to 10 mM in vitro. The study also tested the activity of H_2_S against the effect of two oxidants, namely hydrogen peroxide (H_2_O_2_) and Fe/H_2_O_2_ (a hydroxyl radical donor), on plasma lipids and proteins. The present study used plasma because it is a very important element of hemostasis. Reactive oxygen species (ROS) may induce changes in both the structure and the function of plasma lipids and proteins, which are important hemostatic components and initial targets for ROS. Oxidative modifications of these components have been reported in various conditions, including cardiovascular disorders. The models used in this experiment were similar to the reactions that take place in plasma under oxidative stress conditions. Oxidative stress was measured by examining the concentrations of well-known biomarkers: thiobarbituric acid reactive substance (TBARS), which is a marker of lipid peroxidation; and the concentrations of carbonyl and thiol groups, which are markers of oxidative damage in proteins. 

The toxic and therapeutic effects of H_2_S depend on its concentration. Endogenous concentrations of hydrogen sulfide in human plasma are described within the range 34–65 µM [12]. In the present study, NaHS was administered at concentrations ranging from 0.01 to 10 mM, as these low and high values have also been used in previous studies; Zagli et al. [13] observed that 10 mM NaHS induced the total inhibition of blood platelet aggregation, a very important process in hemostasis, and this inhibition was found to be concentration dependent. Moreover, when fibrinogen was treated with NaHS, even at the highest concentrations of NaHS (1, 5, and 10 mM), it was found to have an inhibitory effect on blood platelet adhesion to modified fibrinogen, a very important hemostatic protein involved in the coagulation process and platelet aggregation [14]. Our earlier results indicate that exogenous H_2_S has anticoagulant properties when administered at physiological concentrations (0.01–100 µM) [15]. As the mechanism(s) behind the relationship between the action of hydrogen sulfide at very high concentrations and hemostasis are still unknown, a secondary aim of our study was to examine the effects of 1, 5, and 10 mM sodium hydrosulfide on the coagulation and fibrinolytic activities of human plasma by determining the maximum velocity of polymerization and the half-life of fibrin lysis. We also examined the amidolytic activity of plasmin in human plasma, and its influence on other hemostatic parameters: the activated partial thromboplastin time (APTT), thrombin time (TT), and international normalized ratio (INR) of human plasma in vitro.

## 2. Materials and Methods

Sodium hydrosulfide, which is known to be a reliable H_2_S donor [16,17], thiobarbituric acid (TBA), 5,5’-dithio-bis-(2-nitrobenzoic acid) (DTNB), and H_2_O_2_ were purchased from Sigma Chemical Co. (Steinheim, Germany). Reagents (for the measurement of hemostasis) were obtained from Diagon Ltd. (Budapest, Hungary) and Boehringer Ingelheim (Ingelheim, Germany). All other chemicals were reagent-grade products purchased from POCh (Gliwice, Poland).

### 2.1. Exposure of Human Plasma to NaHS

Human plasma was obtained from medication-free, regular donors at the blood bank (Lodz, Poland). Plasma was exposed to: (1) NaHS at a final concentration between 0.01 and 10 mM, (2) NaHS at a final concentration between 0.01 and 10 mM plus 2 mM H_2_O_2_, and (3) NaHS at a final concentration between 0.01 and 10 mM plus 4.7 mM H_2_O_2_/3.8 mM Fe_2_SO_4_/2.5 mM EDTA. Samples were incubated for 5, 15, and 30 min at 37 °C (in air-tight tubes). 

### 2.2. Lipid Peroxidation Measurement

Plasma lipid peroxidation was quantified by measuring the concentration of TBARS. The TBARS concentration was calculated using the molar extinction coefficient (ε = 156,000 M^−1^ cm^−1^). More details are described in Wachowicz [18].

### 2.3. Carbonyl Group Measurement

Detection of the carbonyl groups in plasma proteins was carried out according to Dalle-Donne et al. [19]. 2,4-Dintrophenylhydrazine (DNPH) was used for detecting the carbonyl groups. The content of the colored compound was measured spectrophotometrically at 375 nm (using a Helios Alpha UV/Vis Spectrophotometer (Unicam, (Cambridge, UK)). The carbonyl group concentration was calculated based on the molar extinction coefficient (ε = 22,000 M^−1^cm^−1^).

### 2.4. Thiol Group Measurement

The level of the thiol group in plasma proteins was measured spectrophotometrically using a Helios Alpha UV/Vis Spectrophotometer (Unicam) with Ellman’s reagent, i.e., 5,5’-dithiobis-(2-nitrobenzoic) acid (DTNB). The thiol group concentration was calculated using the molar extinction coefficient (ε = 13,600 M^−1^ cm^−1^) [20,21].

### 2.5. The Measurement of Hemostasis Parameters: APTT, TT, INR, Fibrin Polymerization, and Lysis in Plasma

The coagulation times (APTT, TT, and INR) were determined coagulometrically using a K-3002 Optic Coagulation Analyser (Kselmed, Grudziadz, Poland) [22]. Fibrin polymerization and lysis were carried out according to Malinowska et al. [23]. The maximal velocity (V_max_, mOD/min) and maximal absorbance (A_max_) were recorded for each absorbance curve. The half-lysis time was defined as the time needed for the elastic modulus to decline to 50% of its peak value (½A_max_) [23].

### 2.6. Data Analysis

All the values in this study are expressed as means ± SE. The results were analyzed using ANOVA and the Bonferroni post hoc test. In order to eliminate uncertain data, the Q-Dixon test was performed. The level of statistical significance for all tests was taken to be *p* < 0.05.

### 2.7. Statements about Research Involving Human Participants and/or Animals

The protocol of the experiment was approved by the Committee for Research on Human Subjects of the University of Lodz: numbers KBBN-UŁ/I/5/2011 and KBBN-UŁ/II/18/2011.

## 3. Results

**Figure 1:**
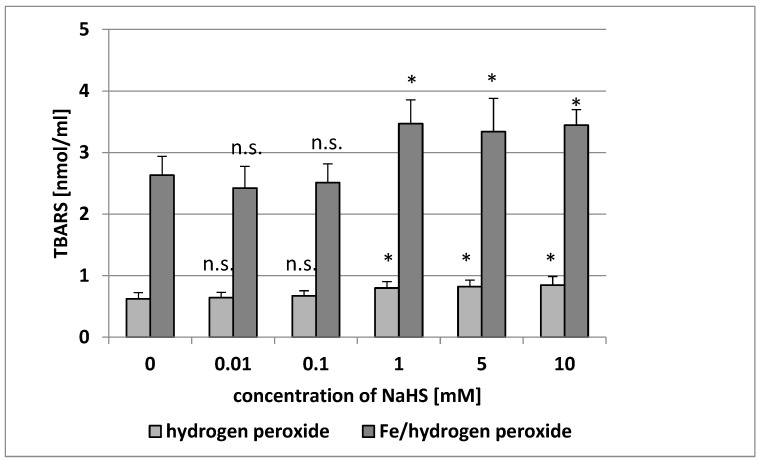
The effect of NaHS (0.01–10 mM, incubation time: 5 min) on plasma lipid peroxidation induced by H_2_O_2_ and by Fe/H_2_O_2_. Data represents the means ± SE of five experiments. *: *p* < 0.05 versus the control (plasma with H_2_O_2_ or Fe/H_2_O_2_), n.s.: *p* > 0.05 versus control (plasma with H_2_O_2_ or Fe/H_2_O_2_). TBARS: thiobarbituric acid reactive substance.

NaHS that was added to human plasma in vitro at concentrations of 0.1–10 mM induced lipid peroxidation (measured using the TBARS level) after 5, 15, and 30 min of incubation (Table 1). The TBARS level increased by about 45% in the presence of 1 mM NaHS after a short incubation time (5 min) compared with the control values (Table 1). However, although the degree of lipid peroxidation of human plasma treated with 0.01 mM NaHS for 5 min was lower than that observed for the controls, this effect was not statistically significant (Table 1). On the other hand, the lipid peroxidation of plasma treated with 0.01 mM for 15 and 30 min was higher than that observed for the control, but for 30 min of incubation, this effect was statistically significant (Table 1). Lipid peroxidation was also enhanced when NaHS was applied at 1–10 mM in human plasma treated with H_2_O_2_ or Fe/H_2_O_2_ (Figure 1). In addition, the lipid peroxidation induced by Fe/H_2_O_2_ was not influenced by NaHS at lower concentrations (0.01 and 0.1 mM) (Figure 1).

**Figure 2:**
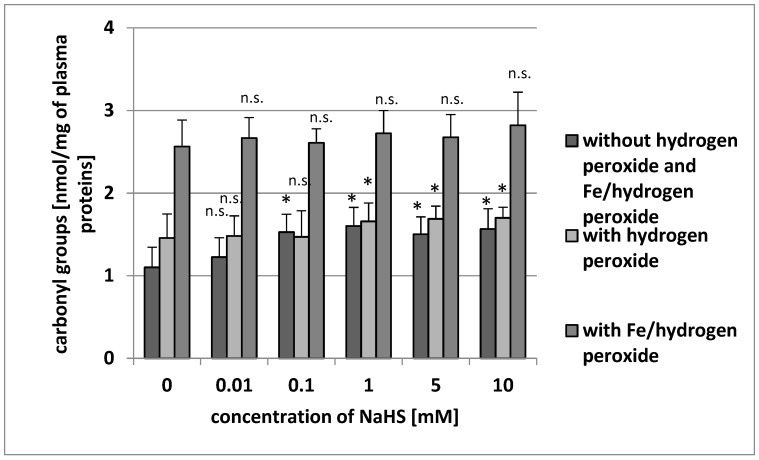
The effect of NaHS (0.01–10 mM, incubation time: 5 min) on carbonyl group formation (plasma protein oxidation) without H_2_O_2_ and Fe/H_2_O_2_, and carbonyl group formation (plasma protein oxidation) induced by H_2_O_2_ and by Fe/H_2_O_2_. Data represents the means ± SE of five experiments. *: *p* < 0.05 versus control, n.s.: *p* > 0.05 versus control.

Our results demonstrate that the level of carbonyl groups was low in human plasma: 1.243 ± 0.269 nmol/mg of plasma proteins. The addition of NaHS (0.1–10 mM) to plasma induced oxidative alterations in proteins as measured by the number of CO groups (Figure 2). NaHS significantly stimulated carbonyl group formation in plasma proteins treated with H_2_O_2_ by about 10% when administered at 1 mM (Figure 2). NaHS (at higher tested concentrations: 5 and 10 mM) also stimulated carbonylation of plasma proteins treated with H_2_O_2_ (Figure 2). On the other hand, none of the tested concentrations of NaHS (0.01–10 mM) was found to change the carbonylation of plasma proteins induced by Fe/H_2_O_2_ (Figure 2).

**Figure 3:**
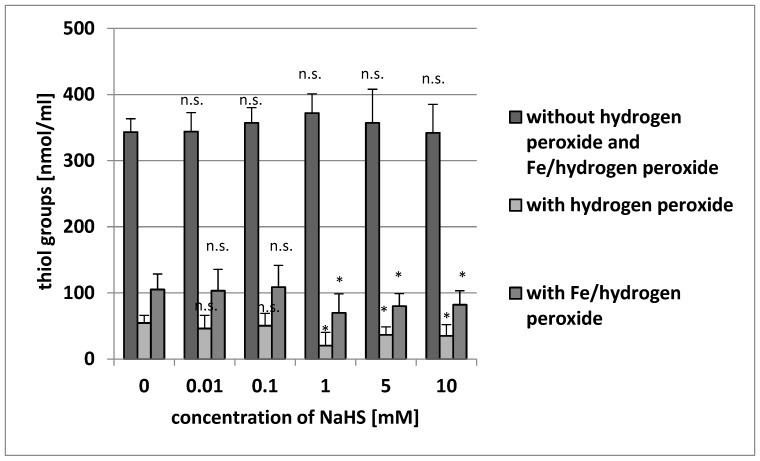
The effect of NaHS (0.01–10 mM, incubation time: 5 min) on the level of thiol groups of plasma proteins (plasma protein oxidation) without H_2_O_2_ and Fe/H_2_O_2_, and the oxidation of protein thiols induced by H_2_O_2_ and by Fe/H_2_O_2_. Data represents the means ± SE of five experiments. *: *p* < 0.05 versus control, n.s.: *p* > 0.05 versus control.

Although NaHS did not change the level of thiol groups in plasma proteins when administered without the addition of H_2_O_2_ or Fe/H_2_O_2_ (*p* > 0.05), a significant decrease was observed in the level of thiol groups in plasma proteins compared with the controls (H_2_O_2_ or Fe/H_2_O_2_) when plasma was incubated with high concentrations of NaHS (1–10 mM) and with H_2_O_2_ or Fe/H_2_O_2_ (Figure 3).

Incubation (5 min) with NaHS (at tested concentrations: 1, 5, and 10 mM) did not induce changes in the coagulation properties of plasma (Table 2). Incubation of human plasma (5 min) with NaHS (1, 5, and 10 mM) did not change APTT, TT, INR (data are not presented), the maximal velocity of fibrin polymerization, or the fibrin lysis in plasma (Table 2). The same process was observed at longer incubation times of 15 and 30 min (data are not presented).

## 4. Discussion

H_2_S may play a role in a range of pathophysiological functions by modulating the oxidative stress observed in various disorders [24]. Only a few studies have demonstrated changes in H_2_S concentrations in human diseases typically associated with oxidative stress, for example, increases have been observed in Down syndrome, septic shock, inflammation of the colon, and diabetes. It is important to note that changes of H_2_S concentration in patients with chronic obstructive pulmonary disease and in smokers may involve oxidative stress. Wang [4,25] reports that hydrogen sulfide may provide protection against hypertension in diabetic patients. Moreover, Azizi et al. [26] suggest that H_2_S may act to reduce oxidative stress. However, the actual concentration of H_2_S in the tested samples was not given; the study simply notes that the level of H_2_S was elevated based on an observed increase in the expression of enzymes involved in H_2_S biosynthesis.

As is the case with its therapeutic action, the toxicity of H_2_S depends on its concentration [27]. Our earlier experiments have demonstrated that in human plasma, the pro-oxidant or antioxidant properties of H_2_S also depend on its concentration, i.e., 0.01 NaHS reduces lipid peroxidation, 0.1 mM NaHS has no effect on this process, and 1 mM NaHS increases it. Moreover, 0.01 and 0.1 mM NaHS was found to decrease plasma lipid peroxidation in a hyperhomocysteinemia model [9].

The present study provides more information on the biological activity of H_2_S in human plasma. Changes in lipid peroxidation, the formation of carbonyl groups, and the oxidation of cysteine residues, demonstrated by the increase of oxidative stress biomarkers, was detected at high concentrations of H_2_S. However, these levels may be toxic and are associated with diseases found to have increases in H_2_S concentration. Our results are the first to demonstrate the pro-oxidant properties of H_2_S on a blood plasma oxidative stress model. The study uses a range of biomarkers to measure oxidative stress: TBARS concentration for lipid peroxidation, and protein carbonyl and thiol groups for oxidative damage in proteins. Protein and lipid auto-oxidation was measured following stimulation by two selected reactive oxygen species: H_2_O_2_ and Fe/H_2_O_2_ (as the donor of OH•).

In plasma (pH 7.4) at 37 °C, 80% of the H_2_S occurs in the form of HS^−^. Through the transfer of a hydrogen atom or single electron, HS^−^ acts as a strong reducing agent that may “extinguish” free radicals [28]. Recently, experiments have demonstrated that H_2_S may react with reactive oxygen species and reactive nitrogen species (RNS), i.e., superoxide radical anion (O_2_^−^•), H_2_O_2_, peroxynitrite (ONOO^−^), or hypochloride. The reaction between H_2_S and ROS/RNS may protect proteins and lipids from oxidation. Inactive nitrozothiols are formed as a result of a chemical reaction between H_2_S and nitric oxide (NO•). Low concentrations of NaHS (30–50 µM) aggravate the protective antioxidative function of glutathione, *N*-acetylcysteine, catalase, superoxide dismutase, and vitamin C.

Studies have demonstrated that H_2_S may decrease the degree of lipid peroxidation, measured as a malondialdehyde (MDA) concentration, and increase the activity of superoxide dismutase in rat hearts subjected to isoproterenol-induced injury [29] and traumatic hemorrhagic shock [30]. Others have found that H_2_S reduced the ROS level in cardiomyocytes under ischemia/reperfusion [31] and in blood platelets [14]. Kimura and Kimura [32] indicate that H_2_S protects neurons from oxidative glutamate toxicity (oxytosis) by increasing the production of glutathione, a very important physiological antioxidant. In addition, it inhibits myocardial injury induced by Hcy in rats [33] and reduces the oxidative stress stimulated by Hcy in vascular smooth muscle cells [34]. However, Hamar et al. [35] suggest that H_2_S is a less effective vascular antioxidant than superoxide dismutase. Morel et al. [14] indicate that NaHS (at different tested concentrations: 0.00001–10 mM) has antioxidant and antiplatelet properties in human blood platelets in vitro. The authors suggest that the antioxidative properties of H_2_S may be associated with its antiplatelet activity on human blood platelets. Our present results indicate that NaHS may have had a pro-oxidative effect at higher concentrations (1, 5, and 10 mM), but this did not change the hemostatic properties of plasma. These results may suggest that H_2_S has different mechanisms of action in blood cells (i.e., platelets) and in plasma.

In the presence of molecular oxygen (O_2_), the auto-oxidation of H_2_S is known to cause the generation of free radicals [27,28]. The present study is the first to describe that 0.1–10 mM NaHS induced the autoperoxidation of human plasma lipids and carbonylation of plasma proteins. Our findings indicate that 1–10 mM NaHS also intensified the lipid peroxidation induced by H_2_O_2_ and Fe/H_2_O_2_. Previous studies have reported that 1–10 mM NaHS has a pro-oxidative effect on the level of thiol groups in plasma proteins treated with H_2_O_2_ and Fe/H_2_O_2_. Wedmann et al. [36] note that NaHS has dramatic actions on protein structure. Our present results indicate that the oxidative protein modifications induced by NaHS may have also stimulated changes in the protein structure. Interestingly, the oxidative stress stimulated by NaHS at high concentrations ranging from 1 to 10 mM was not correlated with changes of hemostasis. The present results are the first to show that high concentrations of H_2_S (1–10 mM) have a different effect on selected elements of hemostasis (coagulation and fibrinolysis) than lower concentrations (0.01–100 µM), which have anticoagulant properties [15]. Incubation (5–30 min) with NaHS at tested concentrations (1, 5, and 10 mM) did not induce changes in the coagulation properties of plasma (Table 2). Our findings indicate that the incubation of human plasma for 5 min with NaHS (1, 5, and 10 mM) did not change APTT, TT, INR, the maximal velocity of fibrin polymerization, or fibrin lysis in plasma (Table 2).

## 5. Conclusions

The obtained results suggest that H_2_S at high concentrations, such as 1 mM, may have a pro-oxidative effect, and that the oxidative stress induced by ROS may be further enhanced by H_2_S. However, the mechanisms behind this pro-oxidative action remain unknown.

## Figures and Tables

**Table 1:** The effect of NaHS (0.01–10 mM, incubation time: 5 min, 15 min, and 30 min) on plasma lipid peroxidation. Data represents means ± SE of five experiments.

Concentration of NaHS (mM)	TBARS (nmol/mL)			
	Incubation Time (min)			
	0	5	15	30
0	0.522 ± 0.099	0.501 ± 0.070	0.488 ± 0.085	0.541 ± 0.059
0.01	0.511 ± 0.077 (n.s. vs. control)	0.489 ± 0.067 (n.s. vs. control)	0.588 ± 0.059 (n.s. vs. control)	0.579 ± 0.059 (*p* < 0.05 vs. control)
0.1	0.499 ± 0.097 (n.s. vs. control)	0.653 ± 0.044 (*p* < 0.001 vs. control)	0.655 ± 0.069 (*p* < 0.05 vs. control)	0.6570 ± 0.062 (*p* < 0.02 vs. control)
1	0.532 ± 0.078 (n.s. vs. control)	0.800 ± 0.099 (*p* < 0.001 vs. control)	0.817 ± 0.089 (*p* < 0.001 vs. control)	0.680 ± 0.087 (*p* < 0.02 vs. control)
5	0.552 ± 0.110 (n.s. vs. control)	0.613 ± 0.111 (*p* < 0.05 vs. control)	0.635 ± 0.080 (*p* < 0.05 vs. control)	0.622 ± 0.116 (*p* < 0.05 vs. control)
10	0.517 ± 0.100 (n.s. vs. control)	0.588 ± 0.079 (*p* < 0.05 vs. control)	0.577 ± 0.071 (*p* < 0.05 vs. control)	0.629 ± 0.096 (*p* < 0.05 vs. control)

**Table 2:** The effect of NaHS on the biological activity of plasma (selected elements of hemostasis). NaHS was preincubated for 5 min at 37 °C with plasma at final concentrations of 1–10 mM.

Concentration of NaHS (mM)	Hemostatic Parameters	
	Fibrin Polymerization V_max_ (% Control)	Fibrin Lysis Time of 50% Lysis (% Control)
0 (control)	100	100
1 mM	98.8 ± 12.5 (n.s.)	104.9 ± 10.3 (n.s.)
5 mM	99.2 ± 15.5 (n.s.)	109.9 ± 13.7 (n.s.)
10 mM	99.7 ± 14.8 (n.s.)	115.8 ± 19.2 (n.s.)
	**Plasmin Amidolytic Activity V_max_ (∆mOD/s)**	
0 (control)	2.057 ± 0.234	
1 mM	2.042 ± 0.183 (n.s.)	
10 mM	1.986 ± 0.176 (n.s.)	

Data represents the means ± SE of 3 experiments (plasmin amidolytic activity) or means ± SE of 10 experiments (for fibrin polymerization and fibrin lysis). n.s.: *p* > 0.05.
